# Effect of nonsurgical periodontal therapy verses oral hygiene instructions on Type 2 diabetes subjects with chronic periodontitis: a randomised clinical trial

**DOI:** 10.1186/1472-6831-14-79

**Published:** 2014-06-25

**Authors:** Renukanth Patabi Cheta Raman, Tara Bai Taiyeb-Ali, Siew Pheng Chan, Karuthan Chinna, Rathna Devi Vaithilingam

**Affiliations:** 1 Department of Restorative Dentistry, Faculty of Dentistry, University of Malaya, 50603 Kuala Lumpur, Malaysia; 2 Department of Oral Biology and Biomedical Sciences, Faculty of Dentistry, University of Malaya, 50603 Kuala Lumpur, Malaysia; 3 Department of Medicine, Faculty of Medicine, University of Malaya, 50603 Kuala Lumpur, Malaysia; 4 Department of Social and Preventive Medicine, Faculty of Medicine, University of Malaya, 50603 Kuala Lumpur, Malaysia

**Keywords:** HbA1c, hs-CRP, Non-surgical periodontal therapy, Oral hygiene instructions, Metabolic control

## Abstract

**Background:**

40 subjects with type 2 diabetes and moderate to severe CP were randomly distributed to groups receiving either NSPT or OHI. Periodontal parameters, glycosylated haemoglobin (HbA1c) and high-sensitivity C-reactive protein (hs-CRP) were evaluated at baseline, 2- and 3-months intervals.

**Methods:**

40 subjects with type 2 diabetes and moderate to severe CP were randomly distributed to groups receiving either NSPT or OHI. Periodontal parameters, glycosylated haemoglobin (HbA1c) and high-sensitivity C-reactive protein (hs-CRP) were evaluated at baseline, 2- and 3-months intervals.

**Results:**

15 subjects from NSPT group and 17 from OHI group completed the study. The difference in plaque index (PI) between NSPT and OHI groups were significant at 2 months recall (p = 0.013). There was no significant difference between NSPT and OHI group for all other clinical periodontal parameters, HbA1c and CRP levels. At 3 months post-therapy, periodontal parameters improved significantly in both groups with sites with probing pocket depth (PPD) < 4 mm reported as 98 ± 1.8% in NSPT group and 92 ± 14.9% in OHI group. Mean PPD and mean probing attachment loss (PAL) within the NSPT group reduced significantly from baseline (2.56 ± 0.57 mm, 3.35 ± 0.83 mm) to final visit (1.94 ± 0.26 mm, 2.92 ± 0.72 mm) (p = 0.003, p < 0.001). For OHI group, improvements in mean PPD and mean PAL were also seen from baseline (2.29 ± 0.69 mm, 2.79 ± 0.96 mm) to final visit (2.09 ± 0.72 mm, 2.62 ± 0.97 mm) (p < 0.001 for both). Similarly, HbA1c levels decreased in both groups with NSPT group recording statistically significant reduction (p = 0.038). Participants who demonstrated ≥ 50% reduction in PPD showed significant reductions of HbA1c and hs-CRP levels (p = 0.004 and p = 0.012).

**Conclusion:**

NSPT significantly reduced PI at 2 months post-therapy as compared to OHI. Both NSPT and OHI demonstrated improvements in other clinical parameters as well as HbA1c and CRP levels.

**Trial registration:**

ClinicalTrials.gov: NCT01951547.

## Background

Periodontal diseases, such as periodontitis, are common infections which if left untreated, can affect the tooth supporting structures and ultimately lead to tooth loss. The main etiological agent of periodontitis is considered to be the dental biofilm [1]. However, multiple causal factors like smoking/tobacco use [2], genetics [3], hormonal changes [4], stress [5], medications [6], diabetes mellitus [7], poor nutrition [8] and systemic diseases [9] that interfere with the body’s immune system play a major role.

Chronic periodontitis has been implicated with several disease processes in the body such as pre-eclampsia and preterm births [10], cardiovascular events [11], respiratory disease [12], chronic renal disease [13], rheumatoid arthritis [14] and diabetes mellitus [7]. Data obtained from several studies strongly suggest diabetes as a risk factor for gingivitis and chronic periodontitis [15]. Evidence also suggests that periodontal changes are the first clinical manifestation of diabetes [16].

Looking from the other perspective, increase in the severity of chronic periodontitis was closely related to the development of glucose intolerance [17]. It has been reported that subjects with severe chronic periodontitis and type 2 diabetes are 6 times more likely to have poorer glycaemic control [18]. Longitudinal studies have reported that infections of periodontal origin have an adverse effect on glycaemic control [19]. A chronic state of hyperglycaemia negatively affects neutrophil function causing a dysfunctional inflammatory response and hampering tissue repair. The concentration of advanced glycation end-products (AGE) which can directly affect normal protein function, or indirectly act by reacting with RAGE (receptors for AGE) on the cell membrane of a variety of cells is elevated in people with type 2 diabetes [20]. These glycated products alter the functional properties of several important matrix molecules such as type 1 collagen and laminin.

The American Academy of Periodontology treatment guidelines [21] stress that periodontal health should be achieved in the least invasive and most cost-effective manner. This is often accomplished through non-surgical periodontal treatment (NSPT), including oral hygiene instructions (OHI), scaling and root planing of the root surface to remove plaque, calculus and bacterial toxins from deep periodontal pockets. Adjunctive therapy, such as antimicrobial therapy and host modulation are incorporated into the treatment regimen as required on a case-by-case basis.

Intervention trials have suggested that there was an improvement in glycaemic control in people with type 2 diabetes following non-surgical periodontal therapy [22]. However, a recent meta-analysis of intervention studies suggests that more evidence is required to confirm the effects of periodontal therapy on the metabolic control of people with type 2 diabetes [23]. The aim of this study was to determine the effect of NSPT as compared to thorough OHI on the metabolic control as well as systemic inflammatory marker, hs C-reactive protein (hs-CRP), among a population of type 2 diabetes afflicted with moderate to severe chronic periodontitis.

## Methods

### Subjects

Ethical approval was obtained from both the Medical Ethics Boards of University of Malaya Medical Centre (MEC Ref No: 696.9) and University of Malaya Dental Centre [DF PE1002/0045(P)]. The study was conducted in accordance with the Helsinki Declaration of 1975, as revised in 2000. The CONSORT guidelines for clinical trials were followed. The Clinical Trials.gov registration number for this study is NCT01951547.

One hundred and twelve people with type 2 diabetes (diagnosed at least 1 year prior to the study) between ages of 30 to 70 were screened from the outpatient Diabetes Clinic of the University of Malaya Medical Centre and a total of 40 subjects who fulfilled the inclusion and exclusion criteria were recruited for the study. The recruited subjects were then registered at the Periodontology Clinic at the Faculty of Dentistry, University of Malaya. Patient information sheets regarding the study and verbal explanations were given to all subjects. Written informed consent was obtained from all recruited subjects with the understanding that they could withdraw from the study at any time. Inclusion criteria were: presence of moderate to advanced chronic periodontitis [24], with at least 12 teeth present and with 5 or more pockets of 5 mm or more and probing attachment loss of 4 mm or more in at least 2 different quadrants which bled on probing. Exclusion criteria included: a history of systemic antibiotic usage over the previous 4 months, having received non-surgical periodontal treatment within the past 6 months or surgical periodontal treatment within the past 12 months; pregnancy, change of medication for diabetes during the course of the study, current smokers or history of a cerebrovascular or cardiovascular event within the past 12 months.

### Sample size calculation

The sample size calculation determined that 15 subjects per treatment arm would provide 80% power to detect a minimum difference of 1% (11.0 mmol/mol) change in HbA1c between test and control [25]. Accordingly, a sample of 20 subjects per arm (40 in total) was recruited to compensate for possible drop-outs during the study period. All subjects were assigned via block randomisation to age matched NSPT and OHI groups [26]. Following randomisation, baseline values for hs-CRP and HbA1c were obtained.

### Examiner calibration

As there was only one examiner involved in the study, intra-examiner reliability assessment was executed. This was done to validate the ability of the examiner to constantly reproduce the quantitative outcome measurements of the clinical parameters used. The Plaque Index (PI) [27], Gingival Bleeding Index (GBI) [27], Probing Pocket Depth (PPD) and Probing Attachment Loss (PAL) were measured in the time interval of about 3 hours. Utilizing Kappa statistics, good agreements (>0.8) were obtained for reproducibility of all recorded clinical parameters.

### Periodontal examination

**Figure 1:**
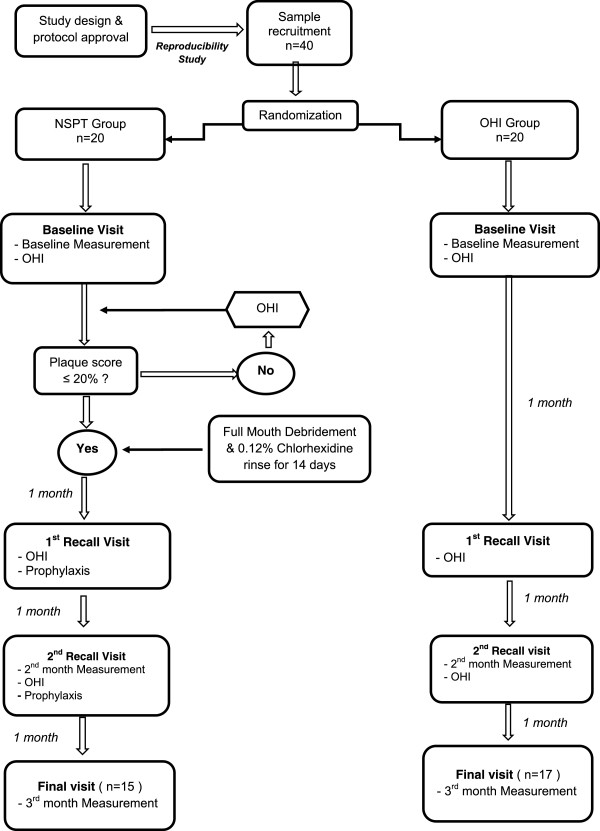
**Flow chart of the Study Protocol.** Flow chart shows the recruitment of subjects and baseline randomization of subjects into the NSPT or OHI groups. Following intervention, subjects are reviewed up to 3 months post-therapy. (OHI: oral hygiene instructions; NSPT: nonsurgical periodontal therapy).

All recruited subjects underwent full periodontal assessment at baseline, 2 months after assigned treatment and 3 months after assigned treatment. The clinical examination included Plaque Index (PI), Gingival Bleeding Index (GBI), Probing Pocket Depth (PPD) and Probing Attachment Loss (PAL) measured with an electronic constant- force probe (Florida Probe®). PPD was measured with the gingival margin as the reference point and PAL was measured with the cement-enamel junction as reference. The trial design and the timing of clinical interventions and sampling are summarized in Figure 1.

### Periodontal intervention

All subjects were instructed in oral hygiene methods using a soft bristled toothbrush, a compact-tuft toothbrush, interdental brushes and dental floss (TePe® oral hygiene education set) utilizing the modified Bass technique. Plaque scores of the subjects in the NSPT group were reviewed at weekly intervals to achieve scores of 20% or below to a maximum of 3 weeks. Subjects were re-motivated and instructed when necessary. Assigned treatment for the NSPT group was full mouth debridement, which consisted of scaling and root planing, was done in a single visit for all subjects in the NSPT group using an ultrasonic scaler (SATELEC P5 Newtron XS, Acteon, Merignac, France) and Gracey curretes (Hu-Friedy, Chicago, USA). Additionally, all subjects in the NSPT group were given a 0.12% Chlorhexidine mouthrinse (Hexipro®, Evapharm, Kuala Lumpur, Malaysia). They were instructed to rinse three times a day using 15 ml each time for a period of 14 days commencing immediately after completion of full mouth debridement. No interventional treatment was given to the OHI group apart from oral hygiene instructions and motivation. Thereafter at each monthly recall visit, participants in both groups were reviewed and re-motivated. Professional prophylaxis comprising of scaling and polishing was performed only on subjects of the test group.

### Measurement of glycated haemoglobin and hs-CRP

15 ml of venous blood was collected from each patient at baseline, prior to treatment and at 3 months after assigned treatments. Levels of glycosylated haemoglobin (HbA1c) and systemic hs-CRP were assessed. Hs-CRP levels were assessed using tests for High sensitivity CRP (hs-CRP). All blood investigations were done at a private laboratory (Gnosis Laboratories Sdn. Bhd) with no affiliation to the Department of Periodontology. The HbA1c testing is DCCT aligned and the Quality Assurance of the laboratory is certified under Bio-Rad Laboratories (USA), EQAS (External Quality Assurance Services).

### Statistical analysis

Comparisons of changes in PI, GBI, PPD (%) and PAL (%) both within and between the groups were performed using the chi-square test. Intragroup comparison for mean PPD, mean PAL, mean HbA1c and mean CRP were assessed with the paired sample t-test whereas intergroup comparisons for the same variables was accomplished using an independent sample t-test.

## Results

Screening and treatment of subjects commenced from May 2010 till April 2011. 15 subjects completed the study protocol in the NSPT group (n = 15) and 17 in the OHI group (n = 17). This gave a within group analyses power of 80% for the NSPT group and 88% for the OHI group. 2 of the 5 subjects from the NSPT group who did not complete the study had their diabetic medication changed during the course of the study and had to be excluded. 2 out of the remaining 3 chose to withdraw from the study for unspecified reasons and the last one was unable to attend recall visits as she was living quite a distance away and was unable to secure transport to the faculty. For the OHI group, 1 subject had his medications for Type 2 diabetes changed and the remaining 2 withdrew consent after the 2 months recall visit for unspecified reasons. Despite the dropouts, the remaining number of subjects in the NSPT and OHI groups was enough to show a power of study of 80%.

At baseline, there was no significant difference between the NSPT and OHI group in terms of age, gender, ethnicity (Table 1) and distribution of periodontal parameters (Tables 2 and 3). All subjects who completed the study were on oral hypoglycaemic drugs.

**Table 1:** Sociodemographic data of study sample

**Variable**	**NSPT group (n = 15)**	**OHI group (n = 17)**	**p-value**
Age (years) (mean ± SD)	57.7 ± 9.9	54.6 ± 6.2	0.28
Ethnicity			
*Malay*	5 (33.3%)	4 (23.5%)	0.75
*Chinese*	4 (26.7%)	4 (23.5%)	
*Indian*	6 (40.0%)	9 (52.9%)	
Gender			
*Male*	11 (73.3%)	9 (52.9%)	0.23
*Female*	4 (26.7%)	8 (47.1%)	
Duration of diabetes			
*< 7 years*	4 (26.7%)	3 (17.6%)	0.76
*7-12 years*	4 (26.7%)	4 (23.5%)	
*> 12 years*	7 (46.7%)	10 (58.8%)	

p-value - independent sample t-test used for age.

-chi-square test used for ethnicity, gender and duration of diabetes.

**Table 2:** Comparison of plaque index and gingival bleeding index at baseline, 2 months and 3 months between NSPT and OHI group

	**NSPT group (n = 15) (% ± SD)**	**OHI group ( n = 17) (% ± SD)**	**p-value**
Plaque index			
Baseline	40.06 ± 22.44	32.70 ± 21.08	0.346
2 months	4.65 ± 4.63	12.06 ± 10.01	0.013*
3 months	1.96 ± 5.97	4.88 ± 5.88	0.174
P_1_ value	<0.001*	<0.001*	
P_2_ value	<0.001*	<0.001*	
P_3_ value	0.157	<0.001*	
Effect Size_1_	1.747	1.480	
Effect Size_2_	1.704	1.577	
Effect Size_3_	0.386	1.193	
Gingival bleeding index			
Baseline	20.81 ± 21.07	21.56 ± 15.22	0.908
2 months	2.97 ± 3.98	6.40 ± 10.00	0.224
3 months	1.18 ± 1.66	14.85 ± 46.32	0.263
P_1_ value	0.006*	<0.001*	
P_2_ value	0.003*	0.565	
P_3_ value	0.092	0.466	
Effect Size_1_	0.844	2.108	
Effect Size_2_	0.942	0.143	
Effect Size_3_	0.467	0.181	

*p < 0.05 ( statistically significant).

p_1_ - p-value of baseline compared to 2 months.

p_2_ - p-value of baseline compared to 3 months.

p_3_ - p-value of 2 months compared to 3 months.

Effect Size_1_ - effect size of baseline compared to 2 months.

Effect Size_2_ – effect size of baseline compared to 3 months.

Effect Size_3_ – effect size of 2 months compared to 3 months.

**Table 3:** Comparison of probing pocket depths and probing attachment loss at baseline, 2 months and 3 months between NSPT and OHI group

	**NSPT group (n = 15)**		**OHI group ( n = 17)**		*p	^**†**^**p**
	**Sites with PPD < 4 mm (% ± SD)**	**Sites with PAL < 4 mm (% ± SD)**	**Sites with PPD < 4 mm (% ± SD)**	**Sites PAL < 4 mm (% ± SD)**		
Baseline	81.14 ± 13.38	63.45 ± 21.29	87.54 ± 15.00	75.98 ± 20.37	0.215	0.099
2 months	95.61 ± 3.50	71.60 ± 19.26	90.32 ± 15.29	78.99 ± 21.17	0.138	0.312
3 month	97.96 ± 1.75	74.93 ± 18.03	91.67 ± 14.86	80.10 ± 21.57	0.633	0.472
P_1_ value	<0.001*	<0.001*	0.001*	<0.001*		
P_2_ value	<0.001*	<0.001*	<0.001*	<0.001*		
P_3_ value	0.008*	0.002*	0.001*	0.005*		
Effect Size_1_	1.205	0.822	0.991	0.792		
Effect Size_2_	1.289	0.982	1.383	0.928		
Effect size_3_	0.794	0.994	0.962	0.786		
	**Sites with PPD**	**Sites with PAL**	**Sites with PPD**	**Sites with PAL**		
	**4-6 mm (% ± SD)**	**4-6 mm (% ± SD)**	**4-6 mm (% ± SD)**	**4-6 mm (% ± SD)**		
Baseline	16.51 ± 10.97	28.62 ± 16.19	11.00 ± 9.52	19.59 ± 12.06	0.201	0.082
2 months	4.28 ± 3.53	23.69 ± 16.84	8.54 ± 10.87	17.55 ± 13.72	0.158	0.264
3 month	2.04 ± 1.75	21.40 ± 16.06	7.31 ± 10.85	16.63 ± 14.44	0.381	0.384
P_1_ value	<0.001*	<0.001*	0.005*	<0.001*		
P_2_ value	<0.001*	<0.001*	<0.001*	<0.001*		
P_3_ value	0.012*	0.013*	0.002*	0.032*		
Effect Size_1_	1.303	0.624	0.797	0.516		
Effect Size_2_	1.371	0.769	1.127	0.619		
Effect Size_3_	0.749	0.733	0.900	0.570		
	**Sites with PPD**	**Sites with PAL**	**Sites with PPD**	**Sites with PAL**		
	**>6 mm (% ± SD)**	**>6 mm (% ± SD)**	**>6 mm (% ± SD)**	**>6 mm (% ± SD)**		
Baseline	2.36 ± 3.14	7.93 ± 7.87	1.54 ± 5.80	4.29 ± 9.77	0.115	0.260
2 months	0.06 ± 0.23	4.55 ± 4.64	1.14 ± 4.69	3.40 ± 8.91	0.074	0.656
3 month	0.00 ± 0.00	3.67 ± 4.06	1.02 ± 4.20	3.27 ± 8.40	0.356	0.869
P_1_ value	0.012*	0.001*	0.168	<0.001*		
P_2_ value	0.012*	0.001*	0.203	<0.001*		
P_3_ value	0.334	0.011*	0.332	0.407		
Effect Size_1_	0.747	0.655	0.350	0.757		
Effect Size_2_	0.749	0.803	0.322	0.612		
Effect Size_3_	0.258	0.751	0.243	0.207		
	**Mean PPD (mean ± SD)**	**Mean PAL (mean ± SD)**	**Mean PPD (mean ± SD)**	**Mean PAL (mean ± SD)**		
Baseline	2.56 ± 0.57	3.35 ± 0.83	2.29 ± 0.69	2.79 ± 0.96	0.535	0.089
2 months	1.94 ± 0.26	2.92 ± 0.72	2.09 ± 0.72	2.62 ± 0.97	0.459	0.329
3 months	1.76 ± 0.19	2.73 ± 0.70	2.02 ± 0.71	2.56 ± 0.97	0.179	0.560
P_1_ value	0.004*	<0.001*	<0.001*	<0.001*		
P_2_ value	0.003*	<0.001*	<0.001*	<0.001*		
P_3_ value	<0.001*	<0.001*	0.001*	0.002*		
Effect Size_1_	0.873	1.893	1.090	1.396		
Effect Size_2_	2.057	2.057	1.528	1.528		
Effect Size_3_	1.506	1.697	0.967	1.282		

*p - intergroup p-value for PPD.

^†^p - intergroup p-value for PAL.

p_1_ - p-value of baseline compared to 2 months (*p < 0.05 - statistically significant).

p_2_ - p-value of baseline compared to 3 months (*p < 0.05 - statistically significant).

p_3_ – p-value of 2 months compared to 3 months (*p < 0.05 -statistically significant).

Effect Size_1_ - effect size of baseline compared to 2 months.

Effect Size_2_ – effect size of baseline compared to 3 months.

Effect Size_3_ – effect size of 2 months compared to 3 months.

### Periodontal parameters

Changes in PI and GBI from baseline to the recall visit (2 months) and to the final visit (3 months) are shown in Table 2. The difference in PI scores between NSPT and OHI groups were significant at 2 months recall (p = 0.013) but by the final visit the difference was no longer significant (p > 0.05). At all time points there were no significant difference in GBI scores between NSPT and OHI groups (p > 0.05).

The reduction in PI scores within the NSPT group from baseline to 2 months recall and final visit were statistically significant (p < 0.001) and the large effect sizes indicated a significant clinical improvement. The reduction in PI seen within the OHI group was also statistically significant (p < 0.001) at all time points. GBI scores for the NSPT group improved from 21% at baseline to 3% at 2 months recall (p = 0.006) and 1.2% at 3 months recall (p = 0.003). For the OHI group, there was an improvement in GBI score from 22% at baseline to 6.4% at recall (p < 0.001). However, at the final visit, the GBI increased 2.5 times, and the change from baseline values were no longer statistically significant (p = 0.466).

At all time points there were no significant difference in mean PPD and mean PAL between NSPT and OHI groups (p > 0.05) (Table 3). The mean PPD within the NSPT group reduced significantly from 6 mm at baseline to 2 mm at recall (p = 0.004) and < 2 mm at the final visit (p = 0.003). The reduction from recall to the final visit was also significant (p < 0.001). In the OHI group, the mean PPD reduced by > 50% at recall (p < 0.001) and reduced further at the final visit (p < 0.001). However between the recall and final visits, the intragroup reductions seen, although significant, were less than the NSPT group. Effect sizes within both groups were large at all time periods, indicating significant clinical changes.

The mean PAL for NSPT group reduced from 3.35 mm at baseline to 2.73 mm at the final visit (Table 3). The changes between all time periods were statistically significant (p < 0.001). In the OHI group, the mean PAL improved from 2.79 mm at baseline to 2.56 mm at the final visit. The changes from baseline to recall and to the final visit were also statistically significant (p < 0.001). The effect size in both groups recorded a considerable improvement in mean PAL during the course of the study. Overall, there were more sites of PPD and PAL of <4 mm at the end of the study and fewer sites with PPD and PAL of > 6 mm.

### Serum HbA1c and CRP levels

At baseline and final visit, HbA1c and CRP levels between NSPT and OHI subjects were not significantly different (p > 0.05) (Table 4). However, within the NSPT group, there was a statistically significant reduction in levels of HbA1c from baseline to final visit (p = 0.038) which was not seen in the OHI group (p = 0.053). For serum CRP levels, the mean value at baseline for the NSPT group was almost twice as high compared to the OHI group but not statistically significant (Table 4). The reductions observed within both the NSPT and OHI groups at the final visit did not reach statistical significance (p > 0.05). However for CRP reduction in the NSPT group, the effect size was moderate which indicated that the improvement was of clinical significance. For the OHI group, the mean CRP at baseline and at the final visit was almost similar.

**Table 4:** Characteristics of monitored systemic markers at baseline and 3 months after treatment

	**NSPT group (n = 15)**	**OHI group (n = 17)**	**p-value**
HbA1c			
% ± SD (mmol/mol ± SD)			
Baseline	7.8 ± 1.5 (61 ± 12)	7.6 ± 1.5 (60 ± 11)	0.791
3 months	7.1 ± 1.2 (54 ± 9)	7.1 ± 1.2 (54 ± 9)	0.995
P value	0.038*	0.053	
Effect Size	0.593	0.495	
hs-CRP			
Baseline	10.5 ± 15.8	5.6 ± 5.2	0.238
3 months	7.0 ± 13.4	5.6 ± 5.3	0.687
P value	0.076	0.783	
Effect size	0.506	0.068	

*p <0.05 ( statistically significant).

### Changes in serum HbA1c and CRP levels for subjects with good periodontal response to treatment

Out of the total sample population, following either NSPT or OHI intervention, participants with a plaque score reduction of ≥ 50% recorded a clinically significant reduction in HbA1c as indicated by the effect size (ES = 0.853) but no significant changes in hs-CRP levels (Table 5). For those participants with a ≥ 50% reduction in gingival bleeding sites, neither HbA1c nor hs-CRP reached statistical significance (p = 0.205 and p = 0.289) but both had large effect sizes (ES = 2.121 and ES = 1.452 respectively), indicating a clinically significant reduction. Out of the total sample population (n = 32), 16 participants demonstrated a ≥ 50% reduction in PPD as an effect of periodontal intervention, be it just OHI or NSPT. These participants also demonstrated a statistically significant reduction in HbA1c (p = 0.004) and hs-CRP (p = 0.012) levels.

**Table 5:** Changes in mean HbA1c and CRP for subjects with good response to periodontal treatment in OHI and NSPT groups

**Reduction of periodontal parameters after treatment**	**Baseline**		**3 months**		**p-value**		**Effect size (ES)**	
	**HbA1c**	**hs-CRP**	**HbA1c**	**hs-CRP**	**p**_**1**_	**p**_**2**_	**ES**_**1**_	**ES**_**2**_
	**(mean ± SD mmol/mol ± SD)**	**(mean ± SD)**	**(mean ± SDmmol/mol ± SD)**	**(mean ± SD)**				
Subjects with a 50% reduction in plaque index scores (n = 7)	8.2 ± 1.1 66 ± 9	5.3 ± 3.6	7.1 ± 1.6 57 ± 12	4.9 ± 3.6	0.065	0.666	0.853	0.171
Subjects with a 50% reduction in gingival bleeding scores (n = 2)	9.0 ± 0.3 75 ± 2	10.0 ± 0.4	7.7 ± 0.4 60 ± 3	8.1 ± 0.9	0.205	0.289	2.121	1.452
Subjects with a 50% reduction in mean PPD (n = 16)	7.7 ± 1.4 60 ± 11	9.6 ± 14.6	6.8 ± 0.8 51 ± 6	8.2 ± 12.9	0.004	0.012	0.859	0.710

p_1_ - p value of HbA1c between baseline and 3 months.

p_2_ - p values of hs-CRP between baseline and 3 months.

ES_1_ - effect size of HbA1c from baseline to 3 months.

ES_2_ - effect size of hs-CRP from baseline to 3 months.

## Discussion

The results of this study demonstrate that in the management of Type 2 diabetic subjects with chronic periodontitis, other than a significant reduction in PI at 2 months post-therapy (p = 0.013), there was no difference in all clinical periodontal parameters, HbA1c and CRP levels when comparing NSPT and OHI. The significant difference in the PI score at 2 months post-therapy could be due to the repeated professional debridement provided as well as the absence of plaque retentive factors in the NSPT group which made plaque control easier and reduced inflammation.

The data also showed clinically significant improvement in periodontal parameters within both groups. Other studies have reported similar findings with the greatest improvements seen in the intervention of test groups [28,29]. Lalla and co-workers [30] recorded a 50% reduction of clinical parameters from baseline. Evidence has always indicated that non-surgical periodontal treatment is the gold standard of periodontal treatment and the results of this study concur. The mere act of proper plaque removal in the OHI group also led to a significant degree of disease resolution, albeit not as quickly or as extensive as seen in the NSPT group. Two prior studies have corroborated this finding with improvements in the plaque index (by as much as 47%) as well as significant reductions in the gingival bleeding index [31,32]. At 3 months post therapy, there was however significant variability in the standard deviation seen for the plaque index in the NSPT group and the gingival bleeding index in the OHI group and this may have been due to the variability in the response to treatment for both groups.

In this study, there was no difference in the improvement in HbA1c levels between NSPT and OHI groups. However, the data indicates that NSPT resulted in a significant improvement in glycaemic control. The NSPT group showed a mean reduction in HbA1c of 0.7% (8 mmol/mol) (p < 0.05) and the OHI group showed a reduction of 0.5% (6 mmol/mol) (p > 0.05). The reduction in HbA1c levels for the NSPT group concur with findings by Darre and colleagues [33] who reported a reduction of 0.8% (9 mmol/mol) following periodontal therapy. Other researchers have also reported the same phenomena [22,34]. HbA1c reductions as high as 17.1% have also been reported [22]. Recently, the team at the Joint EFP/AAP Workshop on Periodontitis and Systemic Diseases published a consensus report stating overwhelming evidence on the effect of periodontal intervention on glycaemic control. Randomized controlled trials (RCTs) consistently demonstrated that mechanical periodontal therapy associates with approximately a 0.4% reduction in HbA1C at 3 months, a clinical impact equivalent to adding a second drug to a pharmacological regime for diabetes [35]. This further affirms the findings of this study as well as those by Sgolastra and colleagues [36] who conducted a meta-analysis on randomized clinical trials on the effect of periodontal treatment on metabolic control. They reported that NSPT was effective at reducing HbA1c and fasting plasma glucose. It is interesting to note that the control group which received OHI alone also demonstrated reductions in their HbA1c levels (p = 0.053, ES = 0.495). Teaching diabetics the proper method of plaque control and correcting improper brushing techniques was able to translate into a clinically relevant improvement in metabolic control.

Both NSPT and OHI demonstrated similar improvements in CRP levels. Improvements in mean levels of hs-CRP in both NSPT and OHI groups did not reach statistical significance. The lack of a statistical significance should be taken with caution as this may be attributed to the small sample size rather than the true difference. Changes in hs-CRP levels were moderate at best and this could be due to the non-specific nature of this inflammatory marker. Reports in the literature have been contradictory [30,37,38]. It has been noted that the reduction in serum hs-CRP was greater in treatment regimens that combine systemic or local antibiotics with standard periodontal treatment [37]. Since no antibiotics were used in this study, it may explain the lack of an association. Another reason for this lack of an association is that during the randomization of subjects, more participants with poor metabolic control were placed in the NSPT group. In the OHI group, there was equal distribution of participants with poor and good metabolic control. Thus the mean CRP levels at baseline were almost 2 fold higher in the NSPT group (10.51) as compared to OHI subjects (5.64). However, at 3 months after treatment, more participants were categorized as well controlled diabetics in both groups. It is important to note that the present data does indicate that participants who responded well to periodontal therapy also recorded clinically relevant reductions in levels of HbA1c and hs-CRP.

Results of this study show that participants who recorded clinically significant improvements in the PI, GBI and PPD also showed concurrent improvement in HbA1c levels. Clinically relevant reductions in CRP levels were also seen in participants with improvements in gingival bleeding scores and PPD. Good periodontal response to interventional periodontal therapy (NSPT or OHI) is mirrored by improvements in metabolic control and reductions in the on-going inflammatory challenge in the body. These results were not in agreement with a study by Llambes et al. [39], who reported no concurrent statistically significant improvements in levels of HbA1c when participants with good periodontal response to treatment were analysed. At the end of the study protocol, there were 16 participants with more than 50% reduction in PPD scores together with statistically significant reduction in HbA1c (p = 0.004) and hs-CRP (p = 0.012) levels. This clearly indicates that re-establishing good periodontal health in type 2 diabetics with periodontal disease has the potential of greatly improving metabolic control and systemic inflammatory challenge. To date, no other study has reported results in this associative manner. However, these findings should be taken with caution as the treatment modalities provided to bring about these improvements come from 2 different intervention methods. A future study with a much larger sample size should be conducted to look at the improvements in periodontal parameters from each intervention method and its effect in improving metabolic control and systemic inflammatory challenge.

The findings from this study show that both NSPT and OHI were able to bring about similar improvements in clinical periodontal parameters as well as HbA1c and CRP levels. Rendering OHI does not require a dental chair or any specific settings, with the right teaching aids it can be taught to anyone. Routine assessment of periodontal health in type 2 diabetes subjects should be performed, and if disease is present, appropriate periodontal management should thus be considered to improve metabolic control and systemic inflammatory challenge. One of the limitations of this study is that this randomized clinical trial was not double-blinded. Due to ethical restrictions, we were unable to have a control group without intervention. A future study which includes double blinding and a much larger sample size would be able to evaluate the potential moderating roles of ethnicity and body weight on metabolic control of these subjects. Also, the moderating role of the chlorhexidine mouthwash needs to be considered, with the inclusion of more study groups that exclude/include the use of the mouthwash.

## Conclusion

NSPT significantly reduced PI scores at 2 months post-therapy as compared to OHI. Both NSPT and OHI demonstrated similar improvements in other clinical parameters as well as HbA1c and CRP levels. Participants who demonstrated ≥ 50% reduction in PPD following either OHI or NSPT, presented with significant reduction in HbA1c and hs-CRP levels.

## Competing interest

The authors declare that they have no conflicts of interest. This study was supported by research grants received from University of Malaya (P0027/2009B and RG344/11HTM].

## Authors’ contribution

RPC Raman was involved in acquisition, analysis and interpretation of data and making the initial draft and revising the manuscript. TB Taiyeb-Ali, RD Vaithilingam and SP Chan made substantial contributions to conception and design of the study, and edited and revised the manuscript. K. Chinna was involved with analysis and interpretation of data and edited/ revised the manuscript. All authors read and approved the final manuscript.

## Pre-publication history

The pre-publication history for this paper can be accessed here:

http://www.biomedcentral.com/1472-6831/14/79/prepub
